# Role of Sonoelastography in Differentiating Benign From Malignant Cervical Lymph Nodes and Correlating With Pathology

**DOI:** 10.7759/cureus.22984

**Published:** 2022-03-09

**Authors:** Eadala Vineela, Anil Kumar Sakalecha, Turuvekere Narayanrao Suresh

**Affiliations:** 1 Radiodiagnosis, Sri Devaraj Urs Academy of Higher Education and Research, Kolar, IND; 2 Pathology, Sri Devaraj Urs Academy of Higher Education and Research, Kolar, IND

**Keywords:** ultrasonography, strain index, elastography pattern, elastography, cervical lymph nodes

## Abstract

Background and aim

Combined use of ultrasonography and elastography improves diagnostic efficacy in differentiating benign from malignant cervical lymph nodes, thereby helping in treatment planning and reducing unnecessary fine needle aspiration cytology/ biopsy. This study aimed to correlate B-mode ultrasonography, color Doppler imaging, and elastography findings with pathological findings and to calculate sensitivity, specificity, and diagnostic accuracy of ultrasonography and elastography.

Material and methods

Patients underwent ultrasonography (B-mode and color Doppler imaging) followed by elastography. Lymph node morphology on B-mode was assessed based on short axis diameter, short-to-long axis ratio, fatty hilum, echogenicity, and margin. Vascularity of lymph nodes on color Doppler imaging was divided into three patterns. On elastography, lymph nodes were defined based on elastography pattern and strain index.

Results

Among all ultrasonography parameters, fatty hilum was found to have the highest diagnostic accuracy (73%), followed by vascularity pattern (70%). Combined use of all ultrasonography parameters yielded better sensitivity (90%), specificity (88%), and diagnostic accuracy (89%) than individual parameters. Five-scale elastography pattern had 83% sensitivity, 97% specificity, and 89% diagnostic accuracy. In the current study, the use of strain index cut-off of two showed sensitivity of 93%, specificity of 96%, and diagnostic accuracy of 94%. Together, ultrasonography and elastography achieved sensitivity of 96%, specificity of 94%, and diagnostic accuracy of 95%.

Conclusion

Elastography can be a useful adjunct to ultrasonography for the accurate diagnosis of cervical lymphadenopathy. Elastography pattern and cut-off strain index of two can effectively differentiate benign from malignant cervical lymph nodes.

## Introduction

Cervical lymphadenopathy can manifest secondary to benign or malignant etiology. Hence, its evaluation will give a clue to the underlying cause and help in treatment planning and ultrasonography (USG) is usually the first preferred imaging modality. Various criteria are defined on B-mode ultrasonography and color Doppler imaging (CDI) for lymph node evaluation. There is however a considerable overlap in diagnostic criteria on B-mode ultrasonography and CDI for differentiating benign and malignant cervical lymph nodes [1,2]. Elastography can help in characterizing lymph nodes based on the stiffness of tissues (malignant tissues are harder than benign). Strain elastography measures the relative stiffness of the lymph node with respect to adjacent normal tissue in response to externally applied manual force [3].

Cervical lymph nodes (CLN) and adjacent normal structures are color-coded representing varying degree of tissue hardness. Elastography pattern is described based on the proportion of blue or hard area on elastograms [4]. Strain index (SI), muscle-to-lymph node strain ratio, is a semi-quantitative measure based on elasticity of the target lymph node with respect to surrounding neck muscles. The probability of malignancy increases as the strain index increases. Elastography can increase the accuracy of ultrasonography in the diagnosis of cervical lymphadenopathy. It can also aid in selecting CLN for fine-needle aspiration cytology (FNAC) which is required for accurate diagnosis and treatment [3,5].

The purpose of this study was to determine whether sonoelastography is an effective tool in differentiating benign from malignant cervical lymph nodes. This study also aimed to correlate B-mode ultrasonography, color Doppler imaging, and elastography findings with pathological findings, and to calculate sensitivity, specificity, and diagnostic accuracy of ultrasonography and elastography.

## Materials and methods

Source of data

The study was conducted over a period of 18 months from January 2019 to June 2020 on 78 patients with enlarged cervical lymph nodes. This study was approved by the institutional ethics committee and informed consent was taken from the patients prior to inclusion in the study. All patients with enlarged CLN who are referred for USG were included in the study. Patients who have received radiotherapy/chemotherapy and underwent recent lymph node FNAC/biopsy were excluded.

Method of collection of data

**Figure 1:**
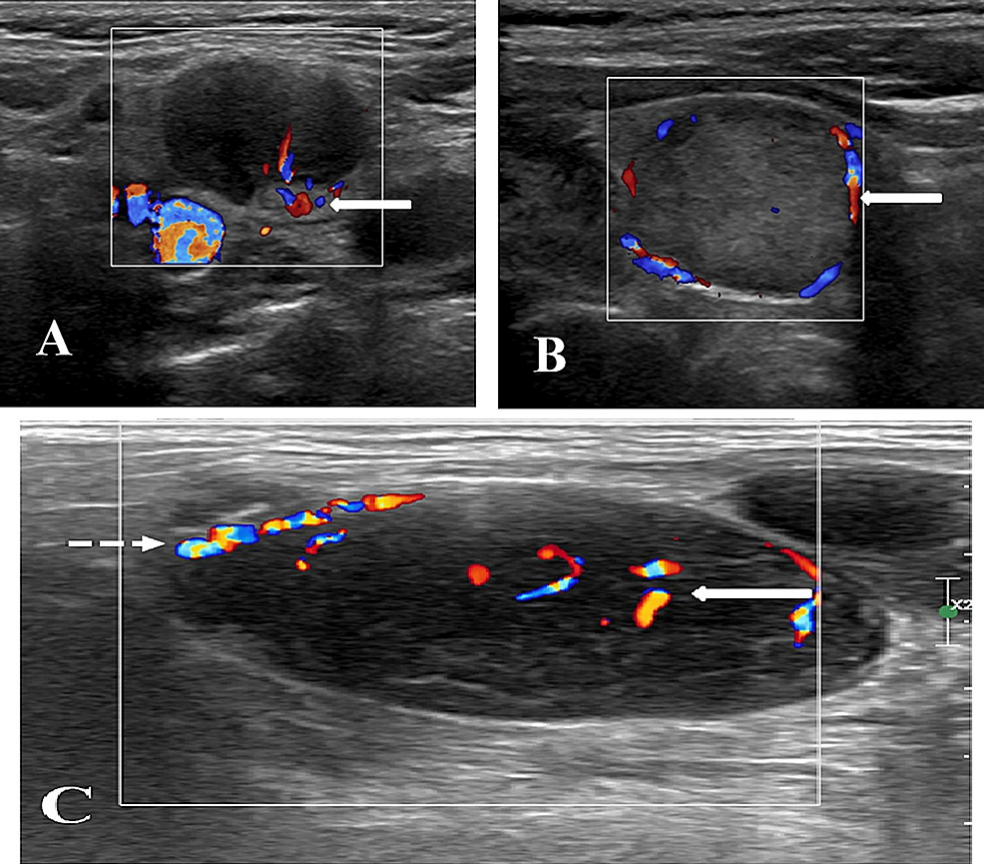
Vascular patterns on color Doppler imaging (A) Pattern 1 shows hilar vascularity (white arrow); (B) pattern 2 shows peripheral vascularity (arrow); and (C) pattern 3 shows mixed vascularity (arrow - central vascularity, dotted arrow - peripheral vascularity). Pattern 1 is a benign pattern and patterns 2 and 3 are malignant patterns.

Informed consent was taken from all the patients before inclusion in the study. Baseline data were collected along with pertinent clinical history, relevant lab investigations, and pathology reports. Individuals with CLN first underwent USG and CDI followed by elastography by using 5-12 MHz linear array transducer (EPIQ 5G ultrasound machine; USA: Philips) by two radiologists (with experience of two and 15 years). Lymph node morphology was defined on USG and vascularity on CDI. B-mode parameters that were evaluated include short-axis dimension (cut-off value: 8 mm), short-to-long axis ratio (cut-off value: 0.6), fatty hilum (presence or absence of hilum), echogenicity (homogenous or heterogenous), and lymph node margin (regular or irregular). Based on the vascularity on CDI, lymph nodes are divided into three patterns - pattern 1 (hilar vascularity or no flow), pattern 2 (peripheral vascularity), and pattern 3 (mixed vascularity). Pattern 1 is considered a benign pattern and patterns 2 and 3 are considered malignant patterns (Figure 1). 

Lymph nodes on elastography were evaluated based on two criteria - elastography pattern and strain index. During strain elastography, a linear probe was placed perpendicularly and gentle compression (> 50%) was applied to generate elastograms (colormaps). Elastography pattern was assessed based on the percentage of blue and red areas in the lymph node. Blue indicates hard area, red indicates soft area, and green indicates intermediate tissue hardness (Table 1).

**Table 1:** Patterns on elastogram for lymph node characterization

Elastography pattern	
Pattern 1	Absent or very small hard or blue area(s).
Pattern 2	Small scattered hard or blue area(s) (< 45%).
Pattern 3	Large hard or blue area(s) (> 45%).
Pattern 4	Peripheral blue area and central green area, suggesting central necrosis.
Pattern 5	Blue area occupying entire lymph node with or without a green or soft rim.

**Figure 2:**
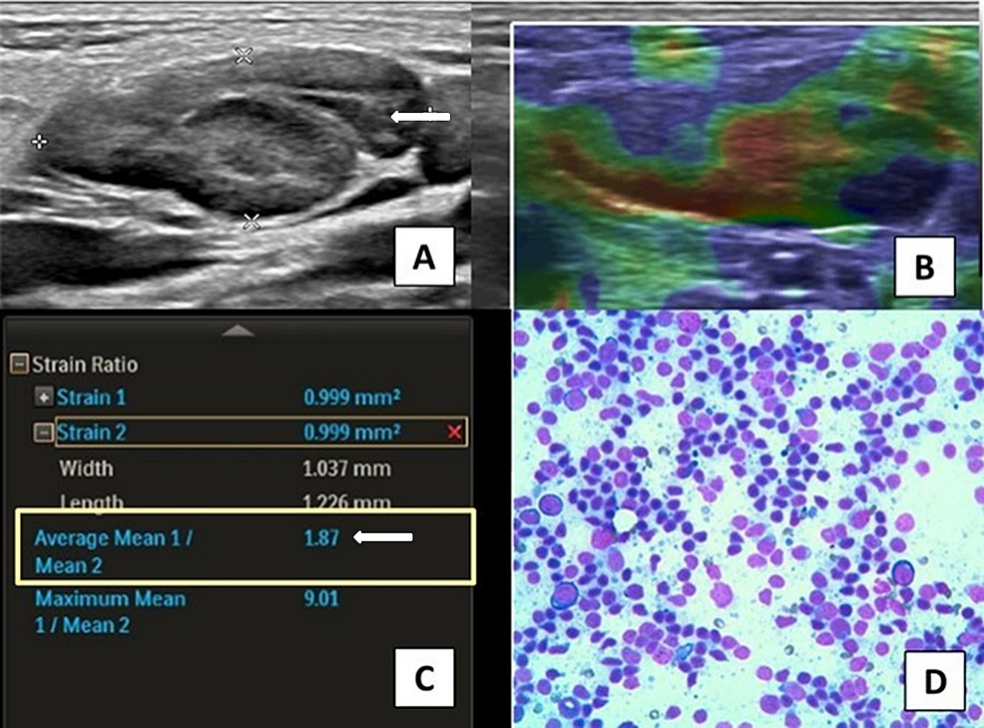
Four-year-old male patient presented with cervical lymphadenopathy (A) On USG, lymph nodes are oval in shape with preserved fatty hilum (arrow). (B and C) On elastography, the lymph node has elastography pattern 2 and SI of 1.87 (arrow). USG and elastography findings are suggestive of benign etiology. (D) FNAC of the lymph node shows reactive cervical lymphadenitis. USG: ultrasonography; SI: strain index; FNAC: fine-needle aspiration cytology

**Figure 3:**
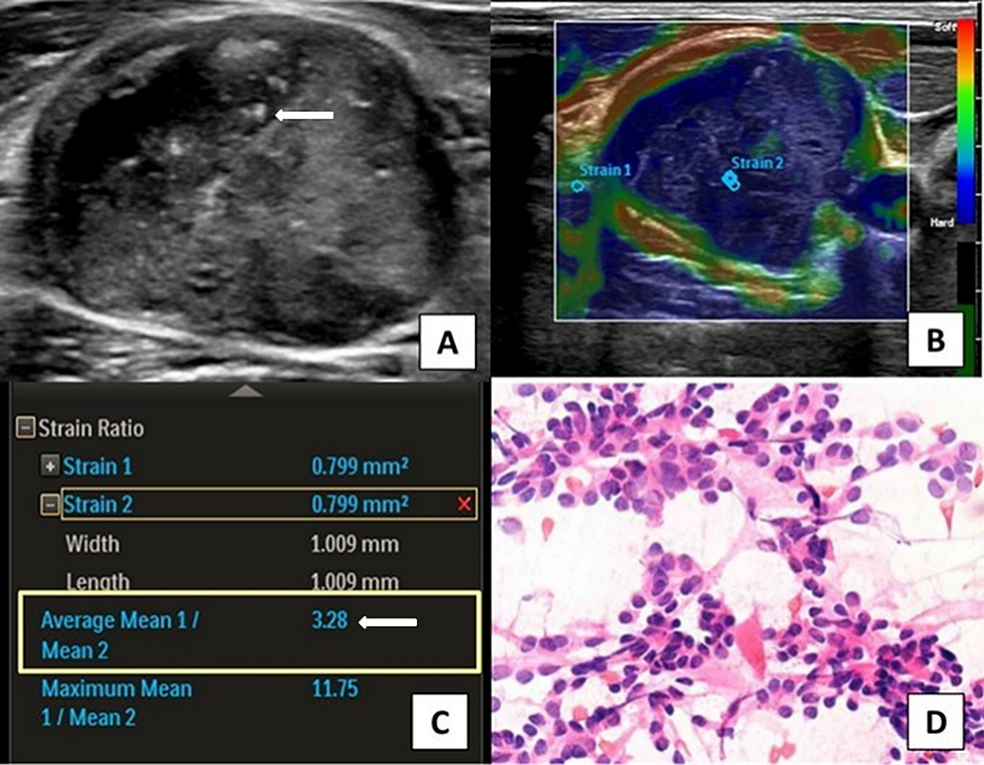
Ultrasound neck of 40-year-old male showing TIRADS 5 lesion in right lobe of thyroid and ipsilateral cervical lymphadenopathy (A) On USG, lymph nodes have multiple intranodal calcifications (arrow) with heterogenous echotexture. (B and C) On elastography, the lymph node shows pattern 5 with SI of 3.28 (arrow). Lymph nodes are diagnosed as malignant based on USG and elastography findings. (D) FNAC of right thyroid lesion and ipsilateral CLN (level 4) show papillary thyroid carcinoma with ipsilateral cervical nodal metastasis. TIRADS: thyroid imaging reporting and data system; USG: ultrasonography; SI: strain index; CLN: cervical lymph nodes; FNAC: fine-needle aspiration cytology

CLN with patterns 1 and 2 were considered as benign and lymph nodes with patterns 3-5 were labeled as malignant lymph nodes. Strain index is the ratio of the hardness of the lymph node with adjacent normal tissue. The first region of interest (ROI 1) was placed in the lymph node and the second region of interest (ROI 2) was placed in the adjacent muscle at the same level. The strain index was calculated as the ratio of ROI 1 to ROI 2 and values were generated. The cut-off strain index used was 2.0. Both USG and elastography findings were recorded and interpreted. The patient underwent pathological investigation, either ultrasound-guided FNAC or biopsy. Elastography and USG findings were compared with pathology findings (Figures 2, 3).

Data analysis

The data were entered in Microsoft excel sheet. The measurable variables were analyzed and interpreted between them by the Student’s t-test and the ordinal and categorical variables between them were interpreted by chi-square (χ^2^) test. The predictive value of strain elastography for differentiating benign and malignant nodes was estimated. The statistical procedures were performed with the help of SPSS version 21.0 (Armonk, NY: IBM. Corp.) and OpenEpi version 3.01. A p-value less than 0.05 was considered statistically significant.

## Results

Demographics

A total of 78 patients were included in our study which was conducted from January 2019 to June 2020. Out of which, benign lymph nodes were 46 and malignant lymph nodes were 32. Demographics are explained eventually in detail in the following subsections.

Age and gender distribution

The mean age in our study was 38 ± 12.8 years with a range of four to 75 years. The commonest age group was 30-59 years (n = 36; 46%). In our study group, there were a total of 43 female (55%) and 35 male (45%) patients. There was a near equal distribution of CLN in male and female patients; 60% of female and 57% of male patients had benign lymph nodes and 40% of female and 43% of male patients had malignant lymph nodes.

B-mode ultrasonography findings

Short-Axis Dimension

Short-axis dimension cut-off value used was 8 mm. A large number of lymph nodes with < 8 mm short-axis dimension were benign. However, out of 63 cervical lymph nodes which had > 8 mm of short axis dimension, 32 were proven as benign and 31 were proven as malignant. Hence, though this parameter has high specificity and statistical significance (p = 0.0026), it has low sensitivity and accuracy (Table 2).

**Table 2:** Short-axis dimension cut-off values PPV: positive predictive value; NPV: negative predictive value

Size of the lymph node	Benign	Malignant	Sensitivity	Specificity	PPV	NPV	Accuracy
< 8 mm	14	1	30 %	97 %	93	49	58 %
> 8 mm	32	31					

Short/Long-Axis Ratio (S/L ratio)

The cut-off value in our study for the short/long-axis ratio was 0.6. S/L ratio of less than 0.6 was found in the majority of benign CLN (87%). However, large number of malignant CLN (63%) also had an S/L ratio of less than 0.6. Though the S/L ratio was statistically significant (p = 0.0116) and had sensitivity of 87%, specificity was low (38%) (Table 3).

**Table 3:** Cut-off values of short/long-axis ratio PPV: positive predictive value; NPV: negative predictive value

Short/long-axis ratio	Benign	Malignant	Sensitivity	Specificity	PPV	NPV	Accuracy
< 0.6	40	20	87%	38%	67	67	67%
> 0.6	6	12					

Lymph Node Margin

A large number (46%) of benign lymph nodes had regular margins. Among benign CLN, suppurative and tubercular etiology nodes were found to have an irregular margin secondary to adjacent inflammation and edema. Out of 32 malignant cervical nodes, 22 nodes showed regular margin and 10 showed irregular margin. Hence, the lymph node margin had poor specificity and was also found to be statistically insignificant (p = 0.152) (Table 4).

**Table 4:** Regular and irregular lymph node margins PPV: positive predictive value; NPV: negative predictive value

	Benign	Malignant	Sensitivity	Specificity	PPV	NPV	Accuracy
Regular	38	22	83%	31%	63	56	62%
Irregular	8	10					

Lymph Node Echogenicity

Benign lymph nodes predominantly showed homogenous echotexture (35 out of 46). However, malignant lymph nodes either showed homogenous (47%) or heterogeneous echogenicity (53%). Statistically significant difference was found in echogenicity of the lymph nodes of benign and malignant etiology (p = 0.0081) (Table 5).

**Table 5:** Homogenous and heterogenous lymph node echogenicity PPV: positive predictive value; NPV: negative predictive value

	Benign	Malignant	Sensitivity	Specificity	PPV	NPV	Accuracy
Homogenous	35	15	76%	53%	70	60	67%
Heterogenous	11	17					

Fatty Hilum

Sixty-seven percent of benign lymph nodes had preserved fatty hila. In the majority (81%) of the malignant lymph nodes, fatty hilum was absent. Among all B-mode parameters, fatty hilum showed high diagnostic accuracy (73%) and a p-value of less than 0.0001 (high statistical significance) (Table 6).

**Table 6:** Presence and absence of fatty hilum in lymph nodes PPV: positive predictive value; NPV: negative predictive value

	Benign	Malignant	Sensitivity	Specificity	PPV	NPV	Accuracy
Present	31	6	67%	81%	84	63	73%
Absent	15	26					

Vascularity on Color Doppler Imaging

Seventy-one percent of benign CLN (33 out of 46 benign lymph nodes) showed vascularity pattern 1 (hilar vascularity or no flow) on CDI, whereas 69% of malignant CLN (22 out of 32 malignant lymph nodes) showed either pattern 2 (peripheral) or pattern 3 (mixed vascularity) on CDI. The vascularity pattern was a statistically significant (p = 0.0004) parameter (Table 7).

**Table 7:** Vascularity patterns of benign and malignant lymph nodes PPV: positive predictive value; NPV: negative predictive value

	Benign	Malignant	Sensitivity	Specificity	PPV	NPV	Accuracy
Benign pattern (1)	33	10	71%	68%	76	62	70%
Malignant pattern (2 and 3)	13	22					

Elastography findings

On elastography, CLN were assessed based on elastography pattern and SI. Elastography findings are further subdivided into elastography pattern and strain index for further explanation.

Elastography Pattern

The maximum number of lymph nodes had pattern 2 on elastography (35 cervical lymph nodes), followed by pattern 3 (18 cervical lymph nodes) and pattern 4 (16 cervical lymph nodes). A total of 39 lymph nodes had benign pattern on elastography (four lymph nodes with pattern 1 and 35 lymph nodes with pattern 2). All four cervical lymph nodes with pattern 1 on elastography were confirmed to be benign on pathology. Of the 35 cervical lymph nodes with pattern 2, 34 lymph nodes were benign and one lymph node was malignant on pathology. There were a total of 39 cervical lymph nodes which showed malignant pattern. Out of these, eight nodes (five lymph nodes with pattern 3 and three lymph nodes with pattern 4) were proven to be of benign etiology and 31 nodes showed malignant features on pathology. All pattern 5 cervical lymph nodes were proven as malignant on pathology. We found a significant difference between the elastography patterns of benign and malignant cervical lymph nodes (p < 0.00001) (Tables 8, 9).

**Table 8:** Distribution of cervical lymph nodes according to elastography pattern

	Total	Benign	Malignant
Pattern 1	4	4	0
Pattern 2	35	34	1
Pattern 3	18	5	13
Pattern 4	16	3	13
Pattern 5	5	0	5

**Table 9:** Distribution of cervical lymph nodes according to elastography pattern

	Benign	Malignant	Sensitivity	Specificity	PPV	NPV	Accuracy
Benign pattern	38	1	83%	97%	97	80	89%
Malignant pattern	8	31					

Elastography Strain Index

We used a mean SI cut-off of 2, which yielded a diagnostic accuracy of 94% and showed statistical significance (p < 0.00001). Among a total of 78 CLN, 44 had SI less than 2, suggestive of benign etiology and 34 lymph nodes had SI more than 2, suggestive of malignant etiology. Mean SI for benign CLN was 1.45 ± 0.306 (mean ± SD), and the mean SI for malignant CLN was 3.01 ± 0.690 (mean ± SD). One malignant lymph node showed SI of less than 2, whereas three benign nodes had SI of more than 2 (Table 10).

**Table 10:** Distribution of cervical lymph nodes according to strain index

	Benign	Malignant	Sensitivity	Specificity	PPV	NPV	Accuracy
Benign (<2)	43	1	93%	96%	97	91	94%
Malignant (>2)	3	31					

Pathology Diagnosis of Cervical Lymph Nodes

**Figure 4:**
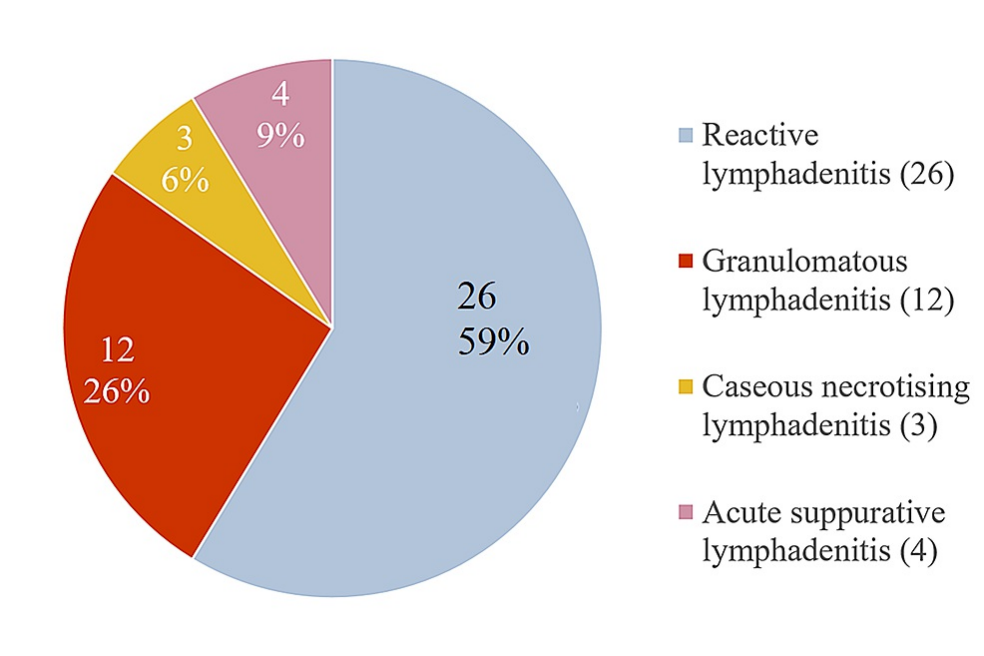
Benign cervical lymph nodes on histopathology

**Figure 5:**
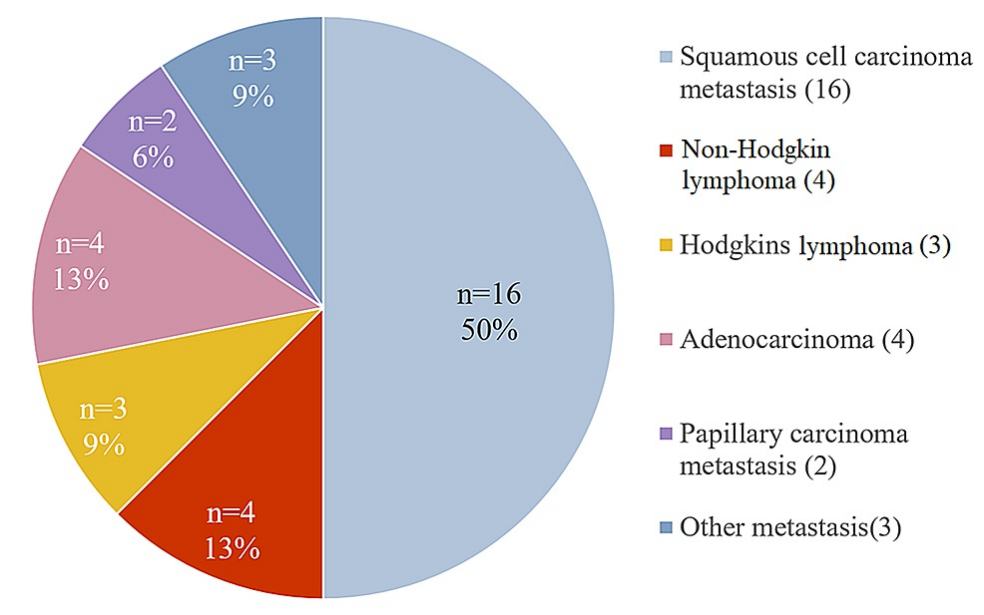
Malignant cervical lymph nodes on histopathology

Following USG and elastography, patient underwent FNAC or biopsy of the lymph node. On final diagnosis, 46 out of 78 (60%) patients had benign lymph nodes and 32 (40%) patients had malignant lymph nodes. Among benign CLN, reactive lymphadenitis (59%) was found to be most common followed by granulomatous lymphadenitis (26%). Among malignant CLN, metastatic lymph nodes accounted for 78% and lymphoma cases accounted for 22%. Metastasis from squamous cell carcinoma of head and neck region (50%) is most common followed by metastasis from adenocarcinoma (13%) (Figures 4, 5).

Comparison of Various Parameters in USG B-Mode, Color Doppler Imaging, and Elastography

All B-mode and CDI parameters except lymph node margin were statistically significant. Among them, fatty hilum and vascularity patterns were found to have high statistical significance and the highest accuracy in differentiating benign from malignant cervical lymph nodes. Both elastography parameters were statistically significant (p < 0.00001) in differentiating cervical lymph nodes. The diagnostic accuracy of the strain index (94%) was marginally better than that of elastography pattern (89%) (Table 11).

**Table 11:** Comparison of USG B-mode, color Doppler, and elastography findings USG: ultrasonography

	Sensitivity (%)	Specificity (%)	Accuracy (%)	p-Value
Short-axis dimension	30	97	58	0.0026
Short/long-axis dimension	87	38	67	0.0116
Echogenicity	76	53	67	0.0081
Margin	83	31	62	0.1520
Fatty hilum	67	81	73	<0.0001
Color Doppler vascularity	71	68	70	0.0004
Elastography pattern	83	97	89	<0.00001
Strain index	93	96	94	<0.00001

Correlation of Ultrasonography and Elastography With Pathological Diagnosis

Our study had 46 benign and 32 malignant lymph nodes diagnosed on pathology. When USG and elastography were used separately, 41 lymph nodes on USG and 43 lymph nodes on elastography were accurately diagnosed as benign; 28 lymph nodes on USG and 30 lymph nodes on elastography were correctly diagnosed as malignant. When both the findings were combined, 44 lymph nodes were accurately diagnosed as benign and 30 lymph nodes were accurately diagnosed as malignant. Combined use of elastography and ultrasonography had augmented diagnostic accuracy (95%) than individual modality (Table 12).

**Table 12:** Correlation of USG and elastography findings with pathological diagnosis USG: ultrasonography

		Pathology diagnosis		Sensitivity	Specificity	Accuracy
		Benign	Malignant			
USG	Benign	41	4	90%	88%	89%
	Malignant	5	28			
Elastography	Benign	43	2	93%	94%	94%
	Malignant	3	30			
USG + elastography	Benign	44	2	96%	94%	95%
	Malignant	2	30			

## Discussion

USG is frequently used as an initial imaging tool for the evaluation of lymphadenopathy. Various etiologies of cervical lymphadenopathy have overlapping imaging features on USG. Pathology is the gold standard for diagnosing the cause, however, it is an invasive procedure. Biopsy/FNAC can be avoided in benign cases, especially in the pediatric population. Elastography can improve the diagnostic efficacy of USG and can aid in decreasing unnecessary FNAC/ biopsy.

Short-axis dimension is the most frequently employed B-mode parameter. Various cut-off values are proposed depending on the location of lymph nodes in the neck. However, a common cut-off value (8 mm) was used by various studies [2,6]. Alam et al. used separate cut-off values for lymph nodes in different neck levels and the diagnostic accuracy was 84%, better than other studies which used common cut-off values [4]. 

Tumor deposit in the lymph node changes the shape from oval to round with resultant increase in S/L ratio. In our study, the S/L ratio showed statistically significant difference between benign and malignant cervical lymph nodes with diagnostic accuracy, sensitivity, and specificity of 67%, 87%, and 38%, respectively. A similar observation was made by Lakshmi et al., who reported a sensitivity of 86.6%, however, they had better specificity than our study [6].

Lymph node margin had a p-value greater than 0.05 (statistically insignificant) and showed poor diagnostic accuracy (62%). An Indian study conducted by Pattanayak et al. concluded that lymph node margin has no statistical significance in differentiating tubercular and metastatic lymph nodes. Irregular nodal border in malignant cases is secondary to extracapsular extension [7]. 

In our study, lymph nodal echogenicity had a diagnostic accuracy of 67%. Similar results were obtained in a study by Abdelgawad et al. [8]. Benign lymph nodes (tuberculous and reactive lymphadenitis) tend to have homogenously hypoechoic echo pattern. However, lymphomatous and metastatic cervical lymph nodes can also be hypoechoic [8,9].

Fatty hilum is one of the commonly used parameters to differentiate benign and malignant cervical lymph nodes. In our study, fatty hilum had the highest diagnostic accuracy (73%) when compared to other B-mode parameters and showed high statistical significance (p < 0.0001). Sensitivity was 67% and specificity was 81%, which is similar to the study conducted by Sathyanarayan and Bharani (75% sensitivity and 84.5% specificity) [10].

Vascularity on CDI showed sensitivity of 71%, diagnostic accuracy of 70%, and specificity of 68%. An Indian study conducted in 2017 on 50 patients also showed results similar to the present study [2]. An Egyptian study by Elzawawy et al. also concluded that hilar vascularity is a benign pattern whereas peripheral and mixed vascularity are malignant patterns [11].

We combined B-mode and CDI findings in the present study. Four cases that were diagnosed as benign on USG were proven as malignant on pathology and five lymph nodes diagnosed as malignant on USG were diagnosed as benign on pathology. Specificity, sensitivity, and diagnostic accuracy were 88%, 90%, and 89%, respectively, which is significantly better than any individual B-mode or color Doppler parameters.

Strain elastography assessment of CLN includes both quantitative and qualitative measurement of the hardness of the lymph node. Elastography pattern is a qualitative assessment based on the percentage of hard area or blue area. In the current study, we used five elastography patterns to characterize lymph nodes on elastogram. Elastography pattern showed 83% sensitivity, 97% specificity, and 89% diagnostic accuracy. Alam et al. were one of the initial researchers to use a five-point scale for elastography pattern and they achieved a diagnostic accuracy of 89% [4]. An Egyptian study conducted in 2017 also used similar elastography pattern scoring and found sensitivity, specificity, PPV, NPV, and diagnostic accuracy of 86%, 100%, 100%, 78.1%, 90.6%, respectively [12].

A four-point scale for elastography pattern was also used in many studies. Four pattern elastography scoring was based on the percentage of blue or hard area, similar to the five-pattern elastography score. Patterns 1 and 2 are considered benign patterns and patterns 3 and 4 are considered malignant patterns [13-15].

In this study, mean SI of 2 was taken as a cut-off value. Among 46 benign CLN, 43 of them showed SI of < 2 and three lymph nodes showed SI of > 2. Out of 32 malignant lymph nodes diagnosed on pathology, 31 had high SI (>2) and only one malignant lymph node had low SI (<2). Difference in the mean SI for benign (1.45 ± 0.306) and malignant (3.01 ± 0.690) CLN was found to be statistically significant. Elastography SI showed higher sensitivity (93%) as compared to elastography pattern (sensitivity of 83%). Specificity (96%) was marginally less than elastography pattern (97%). However, overall diagnostic accuracy of strain index (94%) was better than that of elastography pattern (89%) and all other USG parameters. This observation was consistent with several other studies which compared elastography with B-mode and CDI parameters [1,2,4].

Elastography pattern and SI together had 93% sensitivity, 94% specificity, and 94% diagnostic accuracy. Combined use of elastography and USG gave sensitivity of 96%, specificity of 94%, and diagnostic accuracy of 95%. Several previous studies which compared the diagnostic potential of elastography and ultrasonography reported similar observations [4].

Strain elastography has certain limitations. It can have interobserver and intraobserver variations. We did not evaluate these variations in our study. However, the use of standardized procedure for elastography has improved the reproducibility and diagnostic performance in the current study. Lymphoma lymph nodes tend to be softer and can give low SI. Correlation with B-mode and vascularity pattern can avoid false-negative results.

## Conclusions

In our study, all B-mode parameters (except lymph node margin) and vascularity showed statistically significant difference between benign and malignant CLN. Among them, fatty hilum was found to have the highest diagnostic accuracy (73%), followed by color Doppler vascularity (70%). Combined use of all USG parameters yielded better sensitivity (90%), specificity (88%), and diagnostic accuracy (89%) than individual parameters.

In the current study, the five-scale elastography pattern had diagnostic accuracy of 89%, sensitivity of 83%, and specificity of 97%. SI cut-off of 2 showed sensitivity of 93%, specificity of 96%, and diagnostic accuracy of 94%. Elastography pattern and SI together had sensitivity of 93%, specificity of 94%, and diagnostic accuracy of 94%.

Combined USG and elastography achieved sensitivity of 96%, specificity of 94%, and diagnostic accuracy of 95%. We concluded that elastography can be a useful adjunct to USG. The use of elastography pattern and cut-off SI of 2 along with USG can effectively differentiate benign and malignant CLN, thereby reducing unnecessary invasive procedures and interventions.
